# Supplementary material for: GIVE statistic for goodness of fit in instrumental variables models with application to COVID data
Source: Sci Rep. 2022 Jun 8;12:9472. doi: 10.1038/s41598-022-13240-y (PMC9176169; doi:10.1038/s41598-022-13240-y)
Supplement: Supplementary file 1 — Supplementary Information. [file 41598_2022_13240_MOESM1_ESM.pdf]

Supplement to "GIVE Statistic for Goodness of Fit in Instrumental Variables Models with Application to COVID Data"

Subhra Sankar Dhar[1] & **Shalabh**[2]

Department of Mathematics and Statistics

Indian Institute of Technology Kanpur

Kanpur - 208 016, INDIA

Email-id: [1]subhra@iitk.ac.in, [2]shalab@iitk.ac.in

1 Supplementary Material

1.1 Proofs

Proof of Theorem 1: Note that $\hat{\beta}_{IV}(\hat{\Omega})$ is

$$\begin{aligned} \hat{\beta}_{IV}\hat{\Omega}) - \beta &= (X'P_{Z\hat{\Omega}}X)^{-1}X'P_{Z\hat{\Omega}}(P_{Z\hat{\Omega}}X\beta + u) - \beta \\ &= (X'P_{Z\hat{\Omega}}X)^{-1}X'P_{Z\hat{\Omega}}u. \end{aligned} \tag{1.1}$$

Using (1.1), we have

$$\begin{aligned} (\hat{\beta}_{IV}(\hat{\Omega}) - \beta) &= \left(\frac{X'P_{Z\hat{\Omega}}X}{n}\right)^{-1}\left(\frac{X'P_{Z\hat{\Omega}}u}{n}\right) \\ &\xrightarrow{p} (\Sigma_{XX}^{\Omega})^{-1}.0 \\ &= 0. \end{aligned}$$

It completes the proof. □

Proof of Theorem 2: We first note that

$$\begin{aligned} \beta'X'P_{Z\hat{\Omega}}P_IP_{Z\hat{\Omega}}X\beta &= \beta'\Sigma_{XZ\hat{\Omega}}\Sigma_{ZZ\hat{\Omega}}^{-1}\Sigma_{ZX\hat{\Omega}}\beta \\ &\xrightarrow{p} \beta'\Sigma_{X\Omega X}^{Z}\beta. \end{aligned}$$

Next, consider

$$\begin{aligned}
\left[\hat\beta_{IV}(\hat\Omega)'X'P_{Z\hat\Omega}P_I\hat u_{\hat\Omega}\right] &= \left[\hat\beta_{IV}(\hat{\hat\Omega})'X'P_{Z\hat{\hat\Omega}}P_I(y-P_{Z\hat\Omega}X\hat\beta_{IV}(\hat\Omega))\right] \\
&= \left[\hat\beta_{IV}(\hat\Omega)'X'P_{Z\hat\Omega}P_I(2P_{Z\hat\Omega}X\beta+u-P_{Z\hat\Omega}X\hat\beta_{IV}(\hat\Omega))\right] \\
&= \left[\hat\beta_{IV}(\hat\Omega)'X'P_{Z\hat\Omega}P_IP_{Z\hat\Omega}X\beta\right]+\left[\hat\beta_{IV}(\hat\Omega)'X'P_{Z\hat\Omega}P_Iu\right] \\
&\quad -\left[\hat\beta_{IV}(\hat\Omega)'X'P_{Z\hat\Omega}P_IP_{Z\hat\Omega}X\hat\beta_{IV}(\hat\Omega)\right] \\
&\xrightarrow{p} \beta'\Sigma_{X\Omega Z}\Sigma_{Z\Omega Z}^{-1}\Sigma_{Z\Omega Z}\Sigma_{Z\Omega Z}^{-1}\Sigma_Z\beta+0-\beta'\Sigma_{X\Omega Z}\Sigma_Z^{-1}\Sigma_Z\beta \\
&= 0,
\end{aligned}$$

and

$$\begin{aligned}
(y^{'}P_Iy) &= (P_{Z\hat\Omega}X\beta+u)'P_I(P_{Z\hat\Omega}X\beta+u) \\
&= (\beta'X'P_{Z\hat\Omega}P_IP_{Z\hat\Omega}X\beta+2\beta'X'P_{Z\hat\Omega}P_Iu+u'P_Iu) \\
&\xrightarrow{p} \beta'\Sigma_{XZ\Omega}\Sigma_{ZZ\Omega}^{-1}\Sigma_{ZX\Omega}\beta+0+\sigma^2 \\
&= \beta'\Sigma_{X\Omega X}^{Z}\beta+\sigma^2.
\end{aligned}$$

Thus, we have

$$\begin{aligned}
G_{IV}^2(\hat\Omega) &= 1-\frac{\hat u_{\hat\Omega'P_I\hat u_{\hat\Omega}}}{y'P_Iy} \\
&= \frac{\left(\hat\beta_{IV}(\hat\Omega)'X'P_{Z\hat\Omega}P_IP_{Z\hat\Omega}X\hat\beta_{IV}(\hat\Omega)+2\hat\beta_{IV}(\hat\Omega)'X'P_{Z\hat\Omega}P_I\hat u_{\hat\Omega}\right)}{(y'P_Iy)} \\
&\xrightarrow{p} \frac{\beta'\Sigma_{X\Omega X}^{Z}\beta}{\beta'\Sigma_{X\Omega X}^{Z}\beta+\sigma^2} \\
&= \theta_{IV}(\Omega),\ \ 0\le\theta_{IV}(\Omega)\le 1.
\end{aligned}$$

It completes the proof. □

In order to prove Theorem 3, one needs to prove the following lemma.

Lemma 1 *Under conditions (A1)-(A6),* $\sqrt{n}\{G_{IV}^2(\Omega)-\theta_{IV}(\Omega)\}$ *converges weakly to a normal distribution with mean* $=0$ *and variance* $=\{\nabla g(a)\}^TV\{\nabla g(a)\}|_{a=\beta_{IV}(\Omega)}$.

Proof of Lemma 1: To prove this lemma, we first note that

$$
\begin{aligned}
G_{IV}^2(\Omega) &= \frac{\hat{\beta}_{IV}(\Omega)'X'P_{Z\Omega}(2y - P_{Z\Omega}X\hat{\beta}_{IV}(\Omega))}{y'P_I y} \\
&= \frac{\hat{\beta}_{IV}(\Omega)'X'P_{Z\Omega}(2y - P_{Z\Omega}X\hat{\beta}_{IV}(\Omega))}{(P_{Z\Omega}X\hat{\beta}_{IV}(\Omega) + u)'P_I(P_{Z\Omega}X\hat{\beta}_{IV}(\Omega) + u)},
\end{aligned}
$$

and arguing in a similar way, we have $\theta_{IV}(\Omega) = \frac{\beta_{IV}(\Omega)'X'P_{Z\Omega}(2y - P_{Z\Omega}X\beta_{IV}(\Omega))}{(P_{Z\Omega}X\beta_{IV}(\Omega)+u)'P_I(P_{Z\Omega}X\beta_{IV}(\Omega)+u)}$. In other words, one can write $G_{IV}^2(\Omega) = g(\hat{\beta}_{IV}(\Omega))$ and $\theta_{IV}(\Omega) = g(\beta_{IV}(\Omega))$, where for any $a = (a_1, \ldots, a_d) \in \mathbb{R}^d$ ($d \geq 1$), $g(a) = \frac{a'X'P_{Z\Omega}(2(Xa+u) - P_{Z\Omega}Xa)}{(P_{Z\Omega}Xa+u)'P_I(P_{Z\Omega}Xa+u)}$, i.e., $g : \mathbb{R}^d \to \mathbb{R}$.

Now, it follows from Proposition 2.27 of van der Vaart (1998) that $\sqrt{n}(\hat{\beta}_{IV}(\Omega) - \beta)$ converges weakly to a normal distribution with mean $= 0$ and variance $= V$, where

$$
V = (X'P_{Z\Omega}X)^{-1}(X'P_{Z\Omega}\Omega P_{Z\Omega}X)((X'P_{Z\Omega}X)')^{-1}
$$

. Hence, by delta method (see, e.g., van der Vaart (1998)), one can conclude that $\sqrt{n}(G_{IV}^2(\Omega) - \theta_{IV}(\Omega))$ converges weakly to a Gaussian distribution with mean $= 0$ and variance $= \{\nabla g(a)\}'V\{\nabla g(a)\}|_{a=\beta}$[illegible] where $\nabla g(a) = \left(\frac{\partial g(a)}{\partial a_1}, \ldots, \frac{\partial g(a)}{\partial a_d}\right)$, which completes the proof. □

Proof of Theorem 3: Note that

$$
G_{IV}^2(\hat{\Omega}) = \frac{\hat{\beta}_{IV}(\hat{\Omega})'X'P_{Z\hat{\Omega}}(2y - P_{Z\hat{\Omega}}X\hat{\beta}(\hat{\Omega}))}{y'P_I y}
$$

and

$$
G_{IV}^2(\Omega) = \frac{\hat{\beta}_{IV}(\Omega)'X'P_{Z\Omega}(2y - P_{Z\Omega}X\hat{\beta}(\Omega))}{y'P_I y},
$$

and hence

$$
\begin{aligned}
& \sqrt{n}\{G_{IV}^2(\hat{\Omega}) - \theta_{IV}(\Omega)\} \\
= & \sqrt{n}\{G_{IV}^2(\hat{\Omega}) - G_{IV}^2(\Omega) + G_{IV}^2(\Omega) - \theta_{IV}(\Omega)\} \\
= & \sqrt{n}\{G_{IV}^2(\hat{\Omega}) - G_{IV}^2(\Omega)\} + \sqrt{n}\{G_{IV}^2(\Omega) - \theta_{IV}(\Omega)\}
\end{aligned}
$$

The assertion in Lemma 1 implies that $\sqrt{n}\{G_{IV}^2(\Omega) - \theta_{IV}(\Omega)\}$ converges weakly to a normal distribution with mean $= 0$ and variance $= \{\nabla g(a)\}^T V\{\nabla g(a)\}|_{a=\beta_{IV}(\Omega)}$. So, we now check if $\sqrt{n}\{G_{IV}^2(\hat{\Omega}) - G_{IV}^2(\Omega)\} \xrightarrow{p} 0$ as $n \to \infty$ or not. If this holds true, then the proof is completed.

Further, note that $\hat{\beta}_{IV}(\Omega) = (X'P_{Z\Omega}X)^{-1}X'P_{Z\Omega}y$ and $\hat{\beta}_{IV}(\hat{\Omega}) = (X'P_{Z\hat{\Omega}}X)^{-1}X'P_{Z\hat{\Omega}}y$, as $\hat{\Omega}$ is a consistent estimator of Ω.

Using these expressions and aforementioned fact, we have

$$
\begin{aligned}
& \sqrt{n}\{G^2_{IV}(\hat{\Omega}) - G^2_{IV}(\Omega)\} \\
= & \frac{\sqrt{n}[\hat{\beta}_{IV}(\hat{\Omega})'X'P_{Z\hat{\Omega}}(2y - P_{Z\hat{\Omega}}X\hat{\beta}_{IV}(\hat{\Omega})) - \{\hat{\beta}_{IV}(\Omega)\}'X'P_{Z\Omega}(2y - P_{Z\Omega}X\hat{\beta}_{IV}(\Omega))]}{y'P_Iy} + o_p(1) \\
= & \frac{\sqrt{n}}{y'P_Iy + o_p(1)}[\hat{\beta}_{IV}(\hat{\Omega})'X'P_{Z\hat{\Omega}}2y - \hat{\beta}_{IV}(\hat{\Omega})'X'P_{Z\hat{\Omega}}P_{Z\hat{\Omega}}X\{\hat{\beta}_{IV}(\hat{\Omega})\} \\
& -\{\hat{\beta}_{IV}(\Omega)\}'X'P_{Z\Omega}2y - \{\hat{\beta}_{IV}(\Omega)\}'X'P_{Z\Omega}P_{Z\Omega}X\{\hat{\beta}_{IV}(\Omega)\}] \\
= & \frac{\sqrt{n}}{y'P_Iy + o_p(1)}[\{\hat{\beta}_{IV}(\hat{\Omega}) - \hat{\beta}_{IV}(\Omega) + \hat{\beta}_{IV}(\Omega)\}'X'\{P_{Z\hat{\Omega}} - P_{Z\Omega} + P_{Z\Omega}\}2y \\
& -\{\hat{\beta}_{IV}(\hat{\Omega}) - \hat{\beta}_{IV}(\Omega) + \hat{\beta}_{IV}(\Omega)\}'X'\{P_{Z\hat{\Omega}} - P_{Z\Omega} + P_{Z\Omega}\} \\
& \{P_{Z\hat{\Omega}} - P_{Z\Omega} + P_{Z\Omega}\}X\{\hat{\beta}_{IV}(\hat{\Omega}) - \hat{\beta}_{IV}(\Omega) + \hat{\beta}_{IV}(\Omega)\}] \\
= & \frac{\sqrt{n}}{y'P_Iy + o_p(1)}[\hat{\beta}_{IV}(\Omega)'X'(P_{Z\hat{\omega}} - P_{Z\Omega})2y + \hat{\beta}_{IV}(\Omega)'X'P_{Z\Omega}2y \\
& +\{\hat{\beta}_{IV}(\hat{\Omega})\} - \hat{\beta}_{IV}(\Omega)\}X'\{P_{Z\hat{\Omega}} - P_{Z\Omega}\}2y].
\end{aligned}
$$

Now, note that since the parameter space of β is compact (see (A1)), and $\hat{\sigma}_{i,j} - \sigma_{i,j} = O_p\left(\frac{1}{\sqrt{n}}\right)$ for all i and j (see (A7)), we have $\sqrt{n}(P_{Z\hat{\Omega}} - P_{Z\Omega}) = O_p\left(\frac{1}{\sqrt{n}}\right)$. Moreover, since $\{\hat{\beta}_{IV}(\hat{\Omega})\} - \hat{\beta}_{IV}(\Omega)\} = o_p(1)$, and X and Z are bounded random variables, we have

$$
\frac{\sqrt{n}[\hat{\beta}_{IV}(\Omega)'X'(P_{Z\hat{\omega}} - P_{Z\Omega})2y + \hat{\beta}_{IV}(\Omega)'X'P_{Z\Omega}2y + \{\hat{\beta}_{IV}(\hat{\Omega})\} - \hat{\beta}_{IV}(\Omega)\}X'\{P_{Z\hat{\Omega}} - P_{Z\Omega}\}2y]}{y'P_Iy + o_p(1)} \xrightarrow{p} 0
$$

as $n \to \infty$.

Thus the conditions hold and along with the fact of Lemma 1, it completes the proof.

□

1.2 Application : Multiple Linear Regression Model

The population multiple correlation correlation based on Z_1 and Z_2 are denoted by θ_1 and θ_2, respectively in Tables 1-8 in supplementary files. The results are presented in Tables 1-8

with following notations:

R^2: Coefficient of determination using all the explanatory variables in X,

$G^2_{IV}(Z_1)$: Value of GIVE statistic based on IVs obtained using Wald's method,

$G^2_{IV}(Z_2)$: Value of GIVE statistic based on IVs obtained using Durbins's method

$RB(G^2_{IV}(Z_1))$: Relative bias of $G^2_{IV}(Z_1)$,

$RB(G^2_{IV}(Z_2))$: : Relative bias of $G^2_{IV}(Z_2)$,

$RM(G^2_{IV}(Z_1))$: Relative MSE of $G^2_{IV}(Z_1)$,

$RM(G^2_{IV}(Z_2))$: : Relative MSE of $G^2_{IV}(Z_2)$.

The results in Table 1 are based on $p = 5$ explanatory variables for which the regression coefficients are chosen so that they are not close to zero. The chosen values of regression coefficient are $\beta_1 = 2, \beta_2 = -3, \beta_3 = 5, \beta_4 = -8$ and $\beta_5 = 9$ ensuring thereby that all the variables are relevant and important. The classical coefficient of determination (R^2) has a property that it increases as the number of explanatory variables in the model increases. In order to check this property and to study this behaviour with the proposed GIVE statistics, the number of explanatory variables are increased to $p = 9$. The additional explanatory variables are chosen with respective regression coefficients as $\beta_6 = 7, \beta_7 = -4, \beta_8 = 6$ and $\beta_9 = -7$ which are away from zero ensuring that only the significant explanatory variables are added in the model. The simulation results under this set up are compiled in Table 2. Next we wanted to check the effect of adding the unimportant variables, i.e., those explanatory variables whose corresponding regression coefficients are close to zero, and they are not significantly contributing in explaining the variation in the observations. Therefore, we added four explanatory variables whose regression coefficients are $\beta_6 = 0.07, \beta_7 = -0.004, \beta_8 = 0.06$ and $\beta_9 = -0.0007$, which are close to zero ensuring that the variables added in the model are not significant. The simulation results under this set up are presented in Table 3. The traditional R^2 is defined only when there is an intercept term in the model. To know what happens in GIVE statistics in a model without an intercept term, we consider the set up of Table 2, i.e., $p = 9$ important variables but without an intercept term. The values of GIVE statistics and its simulated results are presented in Table 4.

Table 1: Values, relative bias and relative MSE of G^2, $G^2_{IV}(Z_1)$ and $G^2_{IV}(Z_2)$ for $p = 5$ with all relevant variables under general multiple linear regression model with an intercept term

n	σ^2	θ_1	θ_2	R^2	$G^2_{IV}(Z_1)$	$G^2_{IV}y(Z_2)$	$RB(G^2_{IV}(Z_1))$	$RB(G^2_{IV}(Z_2))$	$RM(G^2_{IV}(Z_1))$	$RM(G^2_{IV}(Z_1))$
50	1.5	0.977	0.984	0.978	0.824	0.843	-0.157	-0.143	0.025	0.021
50	2.5	0.962	0.973	0.940	0.408	0.457	-0.576	-0.531	0.336	0.284
50	3.5	0.936	0.956	0.890	0.172	0.207	-0.816	-0.784	0.669	0.617
50	4.5	0.939	0.956	0.831	0.084	0.100	-0.911	-0.896	0.831	0.804
50	5	0.943	0.957	0.801	0.064	0.075	-0.932	-0.922	0.870	0.851
100	1.5	0.972	0.980	0.977	0.821	0.842	-0.156	-0.142	0.025	0.020
100	2.5	0.956	0.970	0.938	0.395	0.449	-0.587	-0.537	0.347	0.289
100	3.5	0.941	0.958	0.886	0.155	0.194	-0.835	-0.798	0.699	0.638
100	4.5	0.928	0.951	0.825	0.067	0.085	-0.928	-0.911	0.862	0.831
100	5	0.927	0.950	0.793	0.048	0.059	-0.949	-0.938	0.900	0.880
200	1.5	0.978	0.985	0.976	0.819	0.841	-0.162	-0.146	0.027	0.021
200	2.5	0.951	0.966	0.937	0.389	0.445	-0.591	-0.539	0.350	0.291
200	3.5	0.954	0.966	0.884	0.146	0.187	-0.847	-0.806	0.718	0.651
200	4.5	0.931	0.952	0.822	0.058	0.077	-0.937	-0.919	0.879	0.844
200	5	0.920	0.943	0.789	0.039	0.052	-0.958	-0.945	0.917	0.893

Table 2: Values, relative bias and relative MSE of R^2, $G^2_{IV}(Z_1)$ and $G^2_{IV}(Z_2)$ for $p = 9$ with all relevant variables under general multiple linear regression model with an intercept term

n	σ^2	θ_1	θ_2	R^2	$G^2_{IV}(Z_1)$	$G^2_{IV}y(Z_2)$	$RB(G^2_{IV}(Z_1))$	$RB(G^2_{IV}(Z_2))$	$RM(G^2_{IV}(Z_1))$	$RM(G^2_{IV}(Z_1))$
50	1.5	0.983	0.987	0.701	0.849	0.858	-0.136	-0.131	0.019	0.017
50	2.5	0.972	0.979	0.682	0.477	0.506	-0.509	-0.483	0.260	0.234
50	3.5	0.962	0.970	0.655	0.235	0.260	-0.756	-0.732	0.574	0.537
50	4.5	0.951	0.962	0.625	0.127	0.142	-0.866	-0.853	0.752	0.729
50	5	0.946	0.958	0.608	0.101	0.110	-0.893	-0.885	0.799	0.784
100	1.5	0.984	0.990	0.616	0.847	0.863	-0.139	-0.129	0.019	0.017
100	2.5	0.974	0.984	0.602	0.467	0.520	-0.520	-0.472	0.271	0.223
100	3.5	0.964	0.977	0.583	0.214	0.266	-0.778	-0.727	0.607	0.530
100	4.5	0.954	0.971	0.560	0.101	0.131	-0.894	-0.865	0.800	0.749
100	5	0.949	0.968	0.546	0.074	0.095	-0.922	-0.902	0.850	0.814
200	1.5	0.986	0.990	0.568	0.849	0.860	-0.139	-0.131	0.019	0.017
200	2.5	0.976	0.983	0.554	0.471	0.508	-0.518	-0.483	0.268	0.234
200	3.5	0.967	0.976	0.534	0.211	0.249	-0.782	-0.745	0.611	0.555
200	4.5	0.958	0.969	0.510	0.093	0.114	-0.903	-0.882	0.816	0.778
200	5	0.953	0.966	0.496	0.064	0.080	-0.932	-0.917	0.870	0.842

We now analyze the results from Tables 1-3.

It is clear that the random errors in the data affect the values of $G^2_{IV}(Z_1)$ and $G^2_{IV}(Z_2)$. As the value of variance σ^2 increases, the values of $G^2_{IV}(Z_1)$ and $G^2_{IV}(Z_2)$ decrease. The rate

Table 3: Values, relative bias and relative MSE of R^2, $G^2_{IV}(Z_1)$ and $G^2_{IV}(Z_2)$ for $p = 9$ with relevant and unimportant variables under general multiple linear regression model with an intercept term

n	σ^2	θ_1	θ_2	R^2	$G^2_{IV}(Z_1)$	$G^2_{IV}(Z_2)$	$RB(G^2_{IV}(Z_1))$	$RB(G^2_{IV}(Z_2))$	$RM(G^2_{IV}(Z_1))$	$RM(G^2_{IV}(Z_1))$
50	1.5	0.972	0.980	0.974	0.825	0.842	-0.152	-0.141	0.023	0.020
50	2.5	0.954	0.967	0.931	0.414	0.457	-0.566	-0.528	0.323	0.280
50	3.5	0.937	0.955	0.874	0.190	0.218	-0.797	-0.771	0.638	0.597
50	4.5	0.921	0.942	0.808	0.107	0.120	-0.883	-0.873	0.782	0.764
50	5	0.913	0.936	0.774	0.087	0.095	-0.904	-0.898	0.819	0.808
100	1.5	0.973	0.980	0.974	0.820	0.838	-0.157	-0.146	0.025	0.021
100	2.5	0.956	0.968	0.930	0.397	0.440	-0.585	-0.546	0.344	0.299
100	3.5	0.940	0.955	0.873	0.163	0.192	-0.827	-0.799	0.685	0.640
100	4.5	0.924	0.943	0.806	0.077	0.090	-0.916	-0.904	0.840	0.819
100	5	0.916	0.937	0.771	0.059	0.067	-0.936	-0.928	0.877	0.862
200	1.5	0.967	0.978	0.970	0.800	0.828	-0.172	-0.153	0.030	0.024
200	2.5	0.946	0.963	0.920	0.349	0.410	-0.630	-0.574	0.398	0.330
200	3.5	0.925	0.949	0.855	0.126	0.163	-0.863	-0.828	0.746	0.687
200	4.5	0.906	0.935	0.782	0.054	0.069	-0.941	-0.926	0.885	0.857
200	5	0.897	0.929	0.744	0.038	0.049	-0.957	-0.948	0.916	0.898

Table 4: Values, relative bias and relative MSE of R^2, $G^2_{IV}(Z_1)$ and $G^2_{IV}(Z_2)$ for $p = 9$ with all relevant variables under general multiple linear regression model without an intercept term

n	σ^2	θ_1	θ_2	R^2	$G^2_{IV}(Z_1)$	$G^2_{IV}(Z_2)$	$RB(G^2_{IV}(Z_1))$	$RB(G^2_{IV}(Z_2))$	$RM(G^2_{IV}(Z_1))$	$RM(G^2_{IV}(Z_1))$
50	1.5	0.988	0.992	0.686	0.861	0.882	-0.129	-0.111	0.017	0.012
50	2.5	0.981	0.987	0.674	0.515	0.605	-0.475	-0.387	0.226	0.150
50	3.5	0.973	0.981	0.658	0.270	0.404	-0.722	-0.589	0.523	0.347
50	4.5	0.965	0.976	0.637	0.147	0.255	-0.848	-0.739	0.721	0.546
50	5	0.962	0.974	0.625	0.114	0.200	-0.882	-0.794	0.779	0.631
100	1.5	0.980	0.988	0.489	0.837	0.884	-0.146	-0.106	0.021	0.011
100	2.5	0.967	0.980	0.477	0.438	0.613	-0.547	-0.375	0.300	0.141
100	3.5	0.955	0.973	0.458	0.190	0.418	-0.801	-0.570	0.644	0.325
100	4.5	0.943	0.965	0.437	0.089	0.271	-0.905	-0.719	0.820	0.517
100	5	0.937	0.961	0.425	0.067	0.214	-0.929	-0.777	0.863	0.604
200	1.5	0.984	0.989	0.477	0.843	0.882	-0.142	-0.108	0.020	0.012
200	2.5	0.973	0.982	0.466	0.454	0.606	-0.534	-0.383	0.285	0.147
200	3.5	0.962	0.975	0.449	0.196	0.403	-0.796	-0.587	0.635	0.344
200	4.5	0.952	0.969	0.428	0.085	0.251	-0.911	-0.741	0.830	0.550
200	5	0.947	0.965	0.417	0.059	0.193	-0.938	-0.800	0.880	0.641

of such increment depends upon the sample size also. Up to an extent, the larger sample size acts as an antidote for the increasing variance. It is expected that as the value of σ^2 increases, the model fitting becomes bad but the extent of badness is not truly captured through R^2. As σ^2 increases, the values of GIVE statistics decrease much faster than R^2. This confirms and establishes the utility of the proposed GIVE statistics that they are fulfilling the objective for which they were developed. It may be noticed that the values of R^2 are much higher and which may mislead the experimenter that the model is well fitted.

Next, the two choices of instrumental variables, viz., Z_1 and Z_2 are imposed on the same generated data set in every iteration on y and X, and then $G^2_{IV}(Z_1)$ and $G^2_{IV}(Z_2)$ are computed. Therefore, it can be concluded that the difference in the values of $G^2_{IV}(Z_1)$ and $G^2_{IV}(Z_2)$ is due to the difference in the choices of Z_1 and Z_2. It is clearly evident from the Tables 1-3 that $G^2_{IV}(Z_1) < G^2_{IV}(Z_2)$, and all the results indicate that the choice of Z_2 yields a better fitted model than that of Z_1. Such an outcome is intuitively correct also because Z_2 is using more information in terms of ranks of the observations whereas Z_1 is using the information on data as indicator variables only. Hence, it can be concluded that if both Z_1 and Z_2 are used in X, then the proposed GIVE statistics are capable of judging the goodness of fit which is affected primarily due to the choice of IVs and thus deciding over the appropriateness of the choice of IVs. It may be noted that this conclusion is in the situation where all the explanatory variables are replaced by IVs. We have used the same choice of IVs for all the variables but extending it to a case where different explanatory variables are replaced by different IVs is not difficult. The resulting GIVE statistics will reflect the goodness of fit appropriately.

We now consider the relative bias (RB) and relative mean squared error (RM) of $G^2_{IV}(Z_1)$ and $G^2_{IV}(Z_2)$. It is observed from Tables 1-3 that both $G^2_{IV}(Z_1)$ and $G^2_{IV}(Z_2)$ are negatively biased. However, the RB and RM of $G^2_{IV}(Z_2)$ is smaller than that of $G^2_{IV}(Z_1)$ in all the settings of simulation set up. The RB and RM of $G^2_{IV}(Z_1)$ and $G^2_{IV}(Z_2)$ increase as σ^2 increases for the given sample size. As the sample size increases, the RB and RM of $G^2_{IV}(Z_1)$ and $G^2_{IV}(Z_2)$ decrease for a given σ^2. We next consider the results in Tables 1 and 2 and investigate the effect of increasing the significant explanatory variables in the model. It is

clear that the values of $G_{IV}^2(Z_1)$ and $G_{IV}^2(Z_2)$ in Table 2 are higher than their corresponding values in Table 1. This clearly indicates that $G_{IV}^2(Z_1)$ and $G_{IV}^2(Z_2)$ have a tendency that as the the number of explanatory variables increases, the values of $G_{IV}^2(Z_1)$ and $G_{IV}^2(Z_2)$ also increase. This empirically confirms that the values of GIVE statistics increase when explanatory variables are added in the model. This behavior resembles the behaviour of traditional R^2 as in the classical multiple linear regression model.

Now, we investigate what happens to the $G_{IV}^2(Z_1)$ and $G_{IV}^2(Z_2)$ when unimportant explanatory variables are added in the model. Note that the comparison of results in Tables 2 and 3 gives an idea about such behaviour. When relevant explanatory variables are added, we noticed from Tables 1 and 2 then we noticed that both $G_{IV}^2(Z_1)$ and $G_{IV}^2(Z_2)$ increase. Now, consider the results in Tables 2 and 3. We observe that when unimportant explanatory variables are added in the model, then $G_{IV}^2(Z_1)$ and $G_{IV}^2(Z_2)$, both increase with respect to the case of $p = 5$ in Table 1 but it is smaller than the case of $p = 9$ in Table 3. Also, the magnitude of increment is less than the magnitude of increment of $G_{IV}^2(Z_1)$ and $G_{IV}^2(Z_2)$ as in Table 2 where only relevant explanatory variables are added. This clearly indicates that the capability of $G_{IV}^2(Z_1)$ and $G_{IV}^2(Z_2)$ in diagnosing whether the relevant or unimportant explanatory variables are added in the model.

To know if the proposed GIVE statistics require the presence of an intercept term in the model, just like as in the case of traditional R^2, we compare the results in the Tables 2 and 4. The simulation set up in the results in the Tables 2 and 4 are the same and differ only with respect to the intercept term. The results in Table 2 are based on the data generated from a model with an intercept term whereas the results in the Table 4 are based on the data generated from a model without an intercept term. We now compare the results of $G_{IV}^2(Z_1)$ and $G_{IV}^2(Z_2)$ from the Tables 2 and 4. We find that the values of $G_{IV}^2(Z_1)$ and $G_{IV}^2(Z_2)$ increase when the intercept term is absent in the model in comparison to the values of $G_{IV}^2(Z_1)$ and $G_{IV}^2(Z_2)$ when the intercept term is present in the model. There seems no issue that the GIVE statistics do not work in a no intercept term model. It is contrary to the traditional R^2 that is defined only in a model with intercept term. The RBs and RMs of $G_{IV}^2(Z_1)$ and $G_{IV}^2(Z_2)$ are lowered when the intercept term is removed from the

model. Hence, the use of the proposed GIVE statistic in the model without intercept term is recommended. It will give slightly higher values in comparison when the intercept term is present in the model.

We also computed the traditional coefficient of determination (R^2) based on actual explanatory variables to compare its role in the instrumental variable estimation. We computed the traditional R^2 using only the Z_1 and Z_2, i.e., treating Z_1 and Z_2 as if they are the explanatory variable and this is denoted as $R^2(Z_1)$ and $R^2(Z_2)$, respectively along with their RB and RM. The simulated values of $G^2_{IV}(Z_1)$ and $G^2_{IV}(Z_2)$ along with their RB and RM are also computed. The simulated results are presented in Table 5. Analysis of such results will help in understanding the consequences of using the definition of traditional R^2 in IV estimation.

It is observed from Table 5 that though the values of θ_1 and θ_2 are large but R^2 is substantially lower and underestimating them. When Z_1 and Z_2 are used in place of X, then the values of $R^2(Z_1)$ and $R^2(Z_2)$ are again underestimating θ_1 and θ_2, respectively. The degree of underestimation depends heavily on σ^2. The GIVE statistic works well only when σ^2 is small, say less than 1.5 in the given simulation setup. It is intuitively expected that as σ^2 increases, the model fitting should be worsened. The GIVE statistics are capturing it better than R^2. Difference in the values of $G^2_{IV}(Z_1)$ and $G^2_{IV}(Z_2)$ can be interpreted as the difference arising due to the choice of IVs. For example, when $n = 50$, $\sigma^2 = 1.5$, we have $G^2_{IV}(Z_1) = 0.859$ and $G^2_{IV}(Z_2) = 0.866$ whereas $R^2(Z_1) = 0.427$ and $R^2(Z_2) = 0.541$, we can interpret that Z_2 is a better choice than Z_1. As the values of σ^2 increases, the values of $G^2_{IV}(Z_1)$ and $G^2_{IV}(Z_2)$ including R^2, $R^2(Z_1)$ and $R^2(Z_2)$ decrease. This outcome is expected because the variability in the input data becomes high and so any goodness of fit statistics will indicate the bad fitting of the model unless some robust statistical methods are used. Also, The values of R^2 are higher than the values of $R^2(Z_1)$, $R^2(Z_2)$, $G^2_{IV}(Z_1)$ and $G^2_{IV}(Z_2)$. This may be expected because we are replacing a known value by an instrument which will lead to loss of information, and hence, the use of IVs in R^2 and GIVE statistics are expected to decrease. But this behaviour of R^2 is misleading because this indicates that the model is well fitted whereas R^2 is an inconsistent estimator of population multiple

Table 5: Values, relative bias and relative MSE of traditional R^2, $R^2(Z_1)$, $R^2(Z_2)$, $G^2_{IV}(Z_1)$ and $G^2_{IV}(Z_2)$ for $n = 100$ and $p = 9$ with with all relevant variables under general multiple linear regression model with an intercept term

n	σ^2	θ_1	θ_2	R^2	$R^2(Z_1)$	$R^2(Z_2)$	$G^2_{IV}(Z_1)$	$G^2_{IV}(Z_2)$
50	1.5	0.988	0.990	0.551	0.427	0.541	0.859	0.866
50	2.5	0.979	0.984	0.541	0.420	0.531	0.510	0.533
50	3.5	0.971	0.978	0.525	0.408	0.515	0.264	0.289
50	4.5	0.963	0.971	0.508	0.395	0.498	0.143	0.158
50	5	0.960	0.968	0.496	0.387	0.487	0.112	0.123
100	1.5	0.988	0.991	0.519	0.439	0.508	0.857	0.864
100	2.5	0.980	0.984	0.508	0.430	0.497	0.501	0.524
100	3.5	0.973	0.978	0.492	0.417	0.482	0.246	0.272
100	4.5	0.965	0.972	0.473	0.401	0.464	0.119	0.135
100	5	0.962	0.969	0.462	0.392	0.453	0.086	0.098
200	1.5	0.985	0.990	0.498	0.332	0.468	0.848	0.861
200	2.5	0.975	0.983	0.486	0.325	0.457	0.468	0.513
200	3.5	0.966	0.977	0.470	0.315	0.442	0.208	0.255
200	4.5	0.957	0.971	0.450	0.301	0.423	0.091	0.118
200	5	0.952	0.968	0.439	0.294	0.413	0.063	0.082

n	$RB(R^2(Z_1))$	$RB(R^2(Z_2))$	$RB(G^2_{IV}(Z_2))$	$RB(G^2_{IV}(Z_2))$	$RM(R^2(Z_1))$	$RM(R^2(Z_2))$	$RM(G^2_{IV}(Z_1))$	$RM(G^2_{IV}(Z_2))$
50	-0.567	-0.454	-0.130	-0.126	0.322	0.206	0.017	0.016
50	-0.571	-0.460	-0.479	-0.458	0.327	0.213	0.231	0.210
50	-0.580	-0.473	-0.728	-0.704	0.338	0.225	0.533	0.498
50	-0.590	-0.487	-0.852	-0.837	0.350	0.240	0.727	0.702
50	-0.596	-0.497	-0.883	-0.873	0.358	0.250	0.782	0.764
100	-0.556	-0.487	-0.132	-0.128	0.309	0.237	0.018	0.016
100	-0.561	-0.495	-0.489	-0.468	0.315	0.245	0.240	0.219
100	-0.572	-0.508	-0.747	-0.722	0.327	0.258	0.559	0.522
100	-0.584	-0.523	-0.876	-0.861	0.342	0.275	0.769	0.742
100	-0.592	-0.532	-0.910	-0.899	0.352	0.285	0.829	0.809
200	-0.663	-0.528	-0.139	-0.130	0.439	0.279	0.019	0.017
200	-0.667	-0.535	-0.520	-0.478	0.445	0.287	0.271	0.229
200	-0.674	-0.548	-0.784	-0.739	0.455	0.300	0.616	0.547
200	-0.685	-0.564	-0.905	-0.879	0.470	0.319	0.819	0.772
200	-0.691	-0.574	-0.934	-0.915	0.479	0.330	0.872	0.838

correlation coefficient between study and explanatory variables, see Cheng et al. (2014, 2016). For moderately lower values of σ^2, the RB and RM of $G^2_{IV}(Z_2)$ are smaller than that of $R^2(Z_1)$, $R^2(Z_2)$ and $G^2_{IV}(Z_1)$. It is notable that RB and RM of $R^2(Z_1)$, $R^2(Z_2)$, $G^2_{IV}(Z_1)$ and $G^2_{IV}(Z_2)$, as expected, are also high for high values of σ^2.

We also used the expression of asymptotic variance in Theorem 3 to understand its utility in measuring the goodness of fit and choice of instrumental variables. The unknown population parameters were replaced by their respected estimated values that were obtained within the simulation, and the asymptotic variances of $G^2_{IV}(Z_1)$ and $G^2_{IV}(Z_2)$ were computed. However, as expected, the values were highly dependent on the sample size. Also, the values of asymptotic variances depend upon the choice of estimators used for finding the unknown parameter. Nevertheless, the ordering of asymptotic variances was the same as in the case of ordering between $G^2_{IV}(Z_2)$ and $G^2_{IV}(Z_1)$, i.e., the asymptotic variance of $G^2_{IV}(Z_2)$ was smaller than the asymptotic variance of $G^2_{IV}(Z_1)$. Therefore, it can be concluded that although we are recommending to use GIVE statistic but the feasible versions of the asymptotic variance of GIVE statistics can also be used as a criterion for judging the choice of instrumental variables and goodness of fit in IV estimation.

1.3 Application : Measurement Error Model

We first briefly describe the set up of measurement error models. More details can be found in Rao et al. (2008), Cheng and Van Ness (1999). Fuller (1987). Please note that the symbols and notations used in this subsection are limited to this subsection 1.3 only. We have used the standard notations of the measurement error models existing in the literature for easy understanding. The reason to choose the measurement error model for application of GIVE statistics is that the explanatory variable and random errors become correlated when the data is contaminated with the measurement errors. Beside other estimation methods used in measurement error models, the IV method is a popular method to obtain the consistent estimators of the regression coefficient.

We here briefly describe the measurement errors model for better understanding. A basic common assumption of any statistical analysis is that all the observations are correctly observed. In many practical situations, they cannot be correctly observed due to various reasons. They are observed with some measurement error into them. The difference between the observed and true values of the variable is termed as measurement error. We consider

the structural form of the multiple measurement error model where the true explanatory variables are stochastic with the same mean. Let $\eta = (\eta_1, \eta_2, \ldots, \eta_n)$ denote the $(n \times 1)$ vector of observations on the true values of study variable and $T = (t_1, t_2, \ldots, t_n)'$, $t_i = (t_{i1}, t_{i2}, \ldots, t_{ip})'$; $i = 1, 2, \ldots, n$ be the $n \times p$ matrix of the n observations on each of the p explanatory variables which are exactly related as

$$\eta = \alpha e_n + T\beta \tag{1.2}$$

where $\beta = (\beta_1, \beta_2, \ldots, \beta_p)'$ is a $(p \times 1)$ vector of regression coefficients associated with p explanatory variables, α is the intercept term and $e_n = (1, 1, \ldots, 1)'$ is a $(n \times 1)$ vector of elements unity. The true values η and T are not observable due to presence of measurement errors but they are observed as y and X which are $n \times 1$ vector and $n \times p$ matrix, respectively given as

$$y = \eta + \epsilon \tag{1.3}$$

$$X = T + \Delta, \tag{1.4}$$

where $\epsilon = (\epsilon_1, \epsilon_2, \ldots, \epsilon_n)')$ is a $(n \times 1)$ vector of measurement errors in η and $\Delta = (\delta_1, \delta_2, \ldots, \delta_n)'$, $\delta_i = (\delta_{i1}, \delta_{i2}, \ldots, \delta_{ip})'$; $i = 1, 2, \ldots, n$ is $n \times p$ matrix of measurement errors involved in T. We assume that δ_{ij} $(i = 1, 2, \ldots, n,\ j = 1, 2, \ldots, p)$ are independent and identically distributed random variables following $N(0, \sigma_\delta^2)$. Similarly, ϵ_i, $(i = 1, 2, \ldots, n)$ are independent and identically distributed following $N(0, \sigma_\epsilon^2)$. Further, ϵ and Δ are also assumed to be statistically independent of each other.

We follow the same set of parameters to generate the data for simulation as in subsection 1.2. The data is generated from a population with high multiple correlation, $n = 50, 100$ and 200, the measurement errors ϵ and δ are generated following $N(0, \sigma_\epsilon^2 I_n)$ and $N(0, \sigma_\delta^2 I_n)$, respectively with $\sigma_\epsilon^2 = 0.5$, $\sigma_\eta^2 = 1.5, 2.5, 3.5, 4.5$ and 5 for the model (1.2)-(1.4) when $p = 5$ and 9. The observations on T are generated from a normal distribution in every replication a fresh during the simulation, and the corresponding IV's are found to construct Z using the two approaches - Wald Instrument Technique, denoted as Z_1, and Durbin Instrument Technique, denoted as Z_2. The response variable y is then generated, and β is estimated using Z_1 and Z_2 with measurement error ridden data on X and y to further compute the GIVE

statistics. The GIVE statistics based on Z_1 and Z_2 are denoted as $G^2_{IV}(Z_1)$ and $G^2_{IV}(Z_2)$, respectively, and the average of GIVE statistics is computed based on 10000 replications, and the empirical relative bias and empirical relative mean squared error of $G^2_{IV}(Z_1)$ and $G^2_{IV}(Z_2)$ are computed. The population multiple correlation based on Z_1 and Z_2 are denoted by θ_1 and θ_2, respectively. We are not reporting the results when the measurement error variance σ^2_δ in the explanatory variables is increased. Reason being that it does affect the performance of GIVE statistics but it also distorts the input data on X. Obviously, if the input data has too much variation, the statistical tools will surely not behave well. We have chosen the σ^2_δ such that it does not affect the variation in the input data too much.

The simulated results are presented in Tables 6-8. The results in Table 7 are based on $p = 5$ relevant explanatory variables for which the regression coefficients are not close to zero. To study this behaviour of $G^2_{IV}(Z_1)$ and $G^2_{IV}(Z_2)$ by increasing the number of relevant explanatory variables to $p = 9$, the simulation results under this set up are presented in Table 7. Next to check the effect of adding the unimportant variables, four unimportant explanatory variables, whose regression coefficients are close to zero, are added in the model. The outcome of simulation results under this set up are presented in Table 8. The traditional R^2 is not considered as this is an inconsistent estimator of population multiple correlation coefficient, see Cheng et al. (2014, 2016) for more details. The values of β for generating the data are kept the same as in the subsection 1.2.

Now, we analyze the results from Tables 6-8. The conclusions from Tables 6-8 are almost the same as obtained from Tables 1-3.

It is clear that the measurement errors in the data affect the values of $G^2_{IV}(Z_1)$ and $G^2_{IV}(Z_2)$. As the value of variance σ^2_ϵ increases, the values of $G^2_{IV}(Z_1)$ and $G^2_{IV}(Z_2)$ decrease. The rate of such increment depends upon the sample size also. This again confirms that the proposed GIVE statistics can satisfactorily measure the goodness of fit in the measurement error models. The performance of GIVE statistics is good for lower values of σ^2_ϵ and as expected, its values increase as the σ^2_ϵ increases for a given sample size. The performance depends on a combination of n and σ^2_ϵ.

Table 6: Values, relative bias and relative MSE of $G^2_{IV}(Z_1)$ and $G^2_{IV}(Z_2)$ for $p = 5$ with all relevant variables under measurement error model

n	σ^2_ϵ	θ_1	θ_2	$G^2_{IV}(Z_1)$	$G^2_{IV}(Z_2)$	$RB(G^2_{IV}(Z_1))$	$RB(G^2_{IV}(Z_2))$	$RM(G^2_{IV}(Z_1))$	$RM(G^2_{IV}(Z_1))$
50	1.5	0.995	0.996	0.875	0.879	-0.121	-0.117	0.015	0.014
50	2.5	0.992	0.994	0.570	0.590	-0.425	-0.406	0.181	0.165
50	3.5	0.989	0.993	0.339	0.373	-0.657	-0.624	0.434	0.390
50	4.5	0.992	0.994	0.188	0.220	-0.810	-0.779	0.659	0.608
50	5	0.988	0.990	0.139	0.166	-0.859	-0.832	0.739	0.694
100	1.5	0.995	0.997	0.874	0.879	-0.121	-0.118	0.015	0.014
100	2.5	0.991	0.994	0.568	0.589	-0.427	-0.407	0.183	0.166
100	3.5	0.987	0.992	0.331	0.369	-0.665	-0.628	0.443	0.395
100	4.5	0.988	0.991	0.176	0.211	-0.822	-0.787	0.677	0.620
100	5	0.983	0.988	0.127	0.157	-0.871	-0.841	0.759	0.708
200	1.5	0.994	0.996	0.874	0.879	-0.121	-0.118	0.015	0.014
200	2.5	0.990	0.993	0.566	0.588	-0.428	-0.408	0.184	0.166
200	3.5	0.988	0.992	0.327	0.367	-0.669	-0.630	0.448	0.397
200	4.5	0.984	0.990	0.170	0.207	-0.827	-0.790	0.685	0.625
200	5	0.987	0.991	0.121	0.153	-0.877	-0.846	0.770	0.716

Table 7: Values, relative bias and relative MSE of $G^2_{IV}(Z_1)$ and $G^2_{IV}(Z_2)$ for $p = 9$ with all relevant variables under measurement error model

n	σ^2_ϵ	θ_1	θ_2	$G^2_{IV}(Z_1)$	$G^2_{IV}(Z_2)$	$RB(G^2_{IV}(Z_1))$	$RB(G^2_{IV}(Z_2))$	$RM(G^2_{IV}(Z_1))$	$RM(G^2_{IV}(Z_1))$
50	1.5	0.997	0.998	0.882	0.884	-0.116	-0.114	0.013	0.013
50	2.5	0.996	0.997	0.605	0.614	-0.393	-0.384	0.154	0.147
50	3.5	0.991	0.995	0.404	0.424	-0.593	-0.573	0.352	0.329
50	4.5	0.988	0.992	0.257	0.282	-0.740	-0.715	0.549	0.513
50	5	0.993	0.995	0.204	0.228	-0.795	-0.771	0.633	0.596
100	1.5	0.997	0.998	0.881	0.884	-0.116	-0.114	0.013	0.013
100	2.5	0.994	0.997	0.600	0.612	-0.396	-0.386	0.157	0.149
100	3.5	0.995	0.996	0.392	0.418	-0.606	-0.581	0.367	0.337
100	4.5	0.992	0.995	0.240	0.271	-0.758	-0.727	0.576	0.530
100	5	0.990	0.993	0.184	0.214	-0.814	-0.784	0.663	0.616
200	1.5	0.997	0.998	0.881	0.883	-0.116	-0.115	0.014	0.013
200	2.5	0.995	0.997	0.598	0.611	-0.399	-0.387	0.159	0.150
200	3.5	0.994	0.996	0.387	0.415	-0.610	-0.583	0.373	0.340
200	4.5	0.991	0.994	0.231	0.266	-0.767	-0.733	0.588	0.537
200	5	0.992	0.995	0.175	0.208	-0.824	-0.791	0.679	0.626

Tables 6-8 clearly indicate that $G^2_{IV}(Z_1) < G^2_{IV}(Z_2)$ indicating that the choice of Z_2 yields a better fitted model than Z_1. So, it can be concluded that if there are several available choices of IVs, e.g., Z_1 and Z_2 in the present case, then the proposed GIVE statistics helps

Table 8: Values, relative bias and relative MSE of $G^2_{IV}(Z_1)$ and $G^2_{IV}(Z_2)$ for $p = 9$ with relevant and unimportant variables under measurement error model

n	σ^2_ϵ	θ_1	θ_2	$G^2_{IV}(Z_1)$	$G^2_{IV}(Z_2)$	$RB(R^2_{IV}y(Z_1))$	$RB(G^2_{IV}(Z_2))$	$RM(G^2_{IV}(Z_1))$	$RM(G^2_{IV}(Z_1))$
50	1.5	0.995	0.997	0.877	0.880	-0.119	-0.117	0.014	0.014
50	2.5	0.989	0.992	0.579	0.595	-0.414	-0.400	0.172	0.160
50	3.5	0.988	0.992	0.357	0.384	-0.639	-0.613	0.409	0.376
50	4.5	0.985	0.989	0.210	0.236	-0.787	-0.762	0.621	0.582
50	5	0.985	0.990	0.161	0.183	-0.836	-0.815	0.701	0.665
100	1.5	0.995	0.997	0.875	0.879	-0.120	-0.118	0.015	0.014
100	2.5	0.991	0.994	0.572	0.591	-0.423	-0.405	0.179	0.164
100	3.5	0.991	0.993	0.340	0.374	-0.657	-0.623	0.432	0.389
100	4.5	0.985	0.990	0.187	0.220	-0.810	-0.778	0.657	0.606
100	5	0.981	0.987	0.138	0.166	-0.859	-0.832	0.739	0.693
200	1.5	0.995	0.997	0.874	0.879	-0.121	-0.118	0.015	0.014
200	2.5	0.993	0.995	0.568	0.589	-0.428	-0.408	0.183	0.166
200	3.5	0.986	0.990	0.331	0.369	-0.664	-0.627	0.441	0.394
200	4.5	0.983	0.989	0.175	0.211	-0.822	-0.786	0.676	0.619
200	5	0.985	0.989	0.126	0.157	-0.872	-0.842	0.761	0.709

in judging the appropriate choice of IVs and gives an idea about the goodness of the fitted model.

Tables 6-8 reveal that, at least empirically, both $G^2_{IV}(Z_1)$ and $G^2_{IV}y(Z_2)$ are negatively biased. The magnitude of relative bias of $G^2_{IV}(Z_2)$ is smaller than the magnitude of relative bias of $G^2_{IV}(Z_1)$. About the relative mean squared error, we find that $RM(G^2_{IV}(Z_2)) < RM(G^2_{IV}(Z_1))$ in all the settings of simulation set up. One issue we have to keep in mind here is that as the sample size is growing, the values of RB and RM are not necessarily decreasing that much. The reason behind this is that the σ^2_ϵ is also increasing along with the sample size and the impact of σ^2_ϵ is so high that it is compressing the effect of sample size. If we take the sample size to be substantially large, then definitely as the sample size increases, the values of GIVE statistics will converge better towards θ.

We observe that the values of $G^2_{IV}(Z_1)$ and $G^2_{IV}(Z_2)$ in Table 7 are higher than their corresponding values in Table 6. So we can conclude that $G^2_{IV}(Z_1)$ and $G^2_{IV}(Z_2)$ have a tendency to increase as the number of explanatory variables increases. This resembles the behaviour of traditional R^2 in the classical multiple linear regression model which is without

measurement errors. Next, the comparison of results in Tables 7 and 8 indicate that when unimportant explanatory variables are added in the model, then $G^2_{IV}(Z_1)$ and $G^2_{IV}(Z_2)$, both increase but the amount of increment is less than the increment that happened in $G^2_{IV}(Z_1)$ and $G^2_{IV}(Z_2)$ in Tables 7 where only relevant explanatory variables were added. This again confirms the capability of $G^2_{IV}(Z_1)$ and $G^2_{IV}(Z_2)$ in diagnosing whether the relevant or unimportant explanatory variables are added in the model.

Hence, the proposed GIVE statistics works well in the measurement error model. We expect that the GIVE statistics will also work well in other models also.

References

[1] Cheng, C. L. and Van Ness, J. W. (1999) **Statistical Regression with Measurement Error**. London: Arnold and New York: Oxford University Press.

[2] Fuller, W. A. (1987) **Measurement Error Models**. New York: Wiley,

[3] Rao, C. R., Toutenburg, H., Shalabh and Heumann, C. (2008) **Linear Models and Generalizations, Least Squares and Alternatives**, 3rd edition, Springer, Berlin, Heidelberg.

[4] van der Vaart, A. W. (1998) **Asymptotic Statistics**, Cambridge University Press.
